# Applying the Barker School concept of ‘behaviour settings’ to virtual contexts

**DOI:** 10.1098/rstb.2023.0291

**Published:** 2024-08-07

**Authors:** Robert Aunger, Sebastian Deterding, Xiaoyang Zhao, Weston Baxter

**Affiliations:** ^1^ London School of Hygiene & Tropical Medicine, London, UK; ^2^ Dyson School of Design Engineering, Imperial College London, London, UK

**Keywords:** behaviour setting, Roger Barker, mixed reality, virtual reality, conceptual tools

## Abstract

People are spending more and more time interacting with virtual objects and environments. We argue that Roger Barker’s concept of a ‘behaviour setting’ can be usefully applied to such experiences with relatively little modification if we recognize subjective aspects of such experiences such as presence and immersion. We define virtual behaviour settings as virtual environments where the partly or fully digital milieu is synomorphic with and circumjacent to embodied behaviour, as opposed to the fragmented behaviour settings of much-mediated interaction. We present two tools that can help explain and predict the outcomes of virtual experiences—the behaviour setting canvas (BSC) and model—and demonstrate their utility through examples. We conclude that the behaviour setting concept is helpful in both designing virtual environments and understanding their impact, while virtual environments offer a powerful new methodological paradigm for studying behaviour settings.

This article is part of the theme issue ‘People, places, things, and communities: expanding behaviour settings theory in the twenty-first century’.

## 1. Introduction

Much of contemporary human experience is taken up with interactions with virtual objects and spaces (that is, with digitally produced stimuli). We play computer games, do online shopping and read the news on our phones. We already spend 40% of our waking lives online, according to some reports [1]. Many large organizations and governments are actively promoting ‘extended reality’ or ‘metaverse’ technologies and platforms to make virtual or blended virtual/real spaces the default for work and leisure. For this reason, it seems important to determine how to conceptualize, hopefully predict and at times, intervene in behaviour happening in these contexts.

Roger Barker’s concept of a *behaviour setting* is one of the more unusual concepts in the social sciences [2,3]. It is couched neither at the level of the individual nor the population, but in-between—as a description of relatively short-term activity by a relatively small group of interacting people in a circumscribed space. It is highly predictive of human behaviour without referring to complex psychological traits [2,3]. It offers an important way to understand the determinants of human behaviour and how said determinants are situated in a broader context, and represents a powerful tool for supporting design [4–6]. For Barker, behaviour settings describe interdependent standing patterns of behaviour and milieu, where the milieu is *circumjacent* and *synomorphic* with the behaviour pattern. That is, it physically surrounds the behaviour pattern, and milieu and behaviour are structured to mutually fit toward some function. Integrating Barker with other similar situational approaches in social and behavioural research, our own work has spelt out the common kinds of components that can interact and align in a behaviour setting [5]:

— Physical objects: physical attributes of the place where the setting takes place, including its layout (the ‘stage’ for action), objects and infrastructure (e.g. architectural features).
— Social agents: the individuals or groups present within the setting, with a focus on their roles and relationships. These roles influence how individuals behave, communicate and relate to one another within the environment.
— Psychological rules: norms provide a framework for behavioural decision-making and contribute to the establishment of social order and cohesion within the setting. They consist of shared expectations and unwritten rules that govern what happens within the setting.^1^ Psychological competencies may also be required to engage in role performance within certain settings.
— Temporal aspects: the temporal aspect refers to such factors as the duration of the setting, and the timing and sequencing of activities by role-players, summarized as the setting ‘routine’ (which Barker called a ‘standing pattern’). The temporal aspects can influence the pace, organization and coordination of activities within the behaviour setting.

Behaviour settings thus constitute ecological units that integrate ontologically disparate kinds of things—physical, social, psychological and temporal entities—in the same construct, focusing on their functional unity or what Barker called ‘synomorphies’—a high degree of integration between ontologically distinct elements to achieve setting objectives. That is, the form and function of objects define their role in assisting the performance of specific behaviours by agents (e.g. a hammer is designed both to fit in the human hand and to enable increased physical leveraging power to the hand’s motions). Temporal dependencies, physical barriers, enforceable norms, role interactions and required competencies can all interact to constrain the flow of events within a setting.

All that said, Barker did his work before the creation of the internet. Does the explanatory and predictive power of his behaviour set construct transfer to settings that are partly or fully rendered by two-dimensional (2D) screen, mixed reality (MR) and virtual reality (VR) interfaces? That is the question we attempt to explore in this paper. To answer it, our article will advance as follows. First, we will define virtual behaviour settings and distinguish them from fragmented behaviour settings arising from electronic media. We argue that contemporary MR and VR technologies that are identified with terms like ‘metaverse’ re-introduce the tight coupling of behaviour and milieu that have been loosened by prior interactions with fragmented settings, warranting separate treatment. Then, we will identify three pragmatically and phenomenologically distinct forms of virtual behaviour settings: 2D screen-based, MR and VR settings. We will then identify a range of characteristics and potential challenges of virtual environments, and how behaviour setting theory can incorporate these. Next, we introduce two tools we have developed and successfully used in our own work to analyse and intervene in behaviour settings, the behaviour setting model [6] and Behaviour Setting Canvas (BSC) [4]. We will explain how these tools can and have been usefully deployed with virtual behaviour settings. We close with a discussion and outlook on the open research questions and opportunities raised by virtual behaviour settings.

## 2. From fragmented to virtual behaviour settings: why behaviour settings now?

We now take it for granted that people use technology to experience and interact with other people, objects and spaces that are not physically co-present in real time. Yet historically, electronic and then digital media like television and the internet are recent entries into our social fabric that have reorganized it in major ways and continue to do so. Importantly for behaviour setting theory, they dislocate the unity of physical and social aspects of settings [7] that behaviour setting theory takes as a starting point. In today’s ‘permanently online, permanently connected’ [8] world, networked mobile devices like smartphones make the link between the physical setting that surrounds our bodies and the behaviour we engage in ever more tenuous [9]. We can flirt, sell, meet, work, play, learn, diagnose, confess, sit trial, break up, operate heavy machinery and fire guns through our mobile phones while sitting in a bus, doctor’s office or café, walking in a park or lying on a beach. Thus, the immediate physical milieu we bodily inhabit imposes additional and different orders onto the networked, device- and application-based behaviours that now nestle within them (e.g. determining what displays of emotion and voice volume are appropriate when having a business meeting on Zoom in a café). At the same time, our physical spaces are being reshaped to fit the ever-widening variety of behaviours that electronic devices carry into them (e.g. now-ubiquitous wifi and shifting norms about the appropriateness of working with a laptop in a café).

As interesting and important as such *fragmented behaviour settings* are (in which the milieu may be split between two or more physical locations—as in phone calls), they are not the focus of our present analysis. Rather, we are here interested in the current mainstreaming of multi-user virtual and extended reality environments. Enabled by enterprise and consumer MR hardware, platforms like Mozilla Hubs, Meta Horizon, Roblox and similar extended reality applications, we see the rise of virtual behaviour settings that re-introduce a strong link between behaviour and milieu in digital media. With *virtual behaviour settings*, we refer to such instances where the milieu is circumjacent and synomorphic to the behaviour *and* in part or fully constituted by digital stimuli.^2^ That people interact in metaphorical or literal digital spaces is nothing new; these interactions have been subject to intense study in fields like internet research, computer-mediated communication, cyberpsychology, digital sociology, new media studies, games studies, human–computer interaction or presence research for decades. What sets today’s generation of multi-user virtual environments apart is that

— they increasingly aim to carry the whole range of human behaviours (not just play and socializing);
— they do so via richly rendered or augmented circumjacent milieus instead of interfaces embedded within milieus;
— these milieus are often purpose-designed to be synomorphic with specific behaviours; and
— they are becoming mainstreamed (i.e. more common and widespread) and thus enmeshed in the web of other behaviour settings and their material, social, psychological and temporal orders.

All this reinforces the need to consider virtual behaviour-and-circumjacent-milieu units—and the behaviour setting concept offers an analytic lens to understand how this integrated whole functions and can be designed to shape any behaviour in it.

## 3. Types of virtual settings

We distinguish three major pragmatic and phenomenological variants of virtual behaviour settings grounded in underpinning technology: VR, MR and screen-based (2D). Following Milgram and Kishino’s classic reality-virtuality continuum [11], VR is an experience in which an individual engages with a fully computer-mediated world into which no physical objects or agents directly intrude—all perceived agents and objects are either computer-generated simulations or rendered representations of real-world actors and objects. MR describes any set-up that contains both physical and virtual elements; it is an experience that is neither purely real-world nor purely virtual. Adding to Milgram and Kishino, in screen-based or 2D virtual environments (such as Habbo Hotel or Second Life [www.habbo.com/hotel and https://secondlife.com/]), the environment is never fully sensorially circumjacent to the interactants—interaction always occurs via some ‘window’ (i.e. computer screen) into a separate ‘space’.^3^ Table 1 sets out the major differences between these three kinds of experiences.

**Table 1.** Features of the different types of virtual settings.

	2D	MR	VR
ontological status of virtual elements	simulated presence in 2D ‘window’	simulated presence in the real or virtual world	simulated presence in the virtual world
interaction modes with virtual elements	simulated manipulation of avatar- or first-person-based actions via controller	simulated bodily manipulation via info-transmission channel	avatar- or first-person-based activity in virtual space created via device
behaviour setting-based implementation	window as a virtual object simulating a setting running in parallel to real-world setting	virtual objects/infrastructure added to real-world setting model	virtual world as virtual setting model running parallel to real-world setting

## 4. Conceptual challenges and characteristics of virtual settings

As internet research and other fields tell us, the physical and social features of virtual spaces need not have the same constraints or affordances as the real world. Virtual spaces allow agents to manipulate and transcend the laws of physics, allowing flight, superhuman feats and other ‘magical interactions’ [12]. That said, the range of sensory stimuli possible depends on the capacities of the physical interface technology. Commercial interfaces today are primarily visual and auditory.

Social dynamics can also differ. For instance, avatars allow participants to appear in different bodies, interpersonal cues are selectively ‘filtered out and in’, and anonymity and lack of direct bodily exposure to interaction partners can invite norm-breaking or ‘toxic’ behaviour [13,14].

On the psychological front, social norms may not be shared with or towards virtual interactants, especially if they comprise human and AI actors (Zhao [15]). Where norms exist, the mechanisms for reinforcement have varying levels of temporal response and intensity. The potential fleetingness and rapid inflow and outflow of large numbers of new interactants can make maintaining norms in virtual settings challenging [16].

In the following, we unpack several aspects worthy of particular note to behaviour setting theory with regard to virtual behaviour settings: objects and spaces, avatar embodiment, behaviour and presence.

### (a) Virtual objects and spaces

Virtual environments render everyday physical constraints such as solid obstacles and laws like gravity malleable by the designer—and/or user, if designers grant this [17]. Where real-world objects more or less obdurately resist change (often requiring time and multiple interactions), virtual objects can be created, copied, deleted or modified instantly and seemingly without cost. Furthermore, agents typically manipulate virtual objects indirectly, through digital interfaces or controllers, which may involve gestures, button presses or other input methods, with consequences that need not resemble their physical counterparts (e.g. controller vibration).

Virtuality can further layer and link additional information and functionality onto objects, places or people in the environment. For example, in a museum setting, MR can provide detailed information about exhibits or artefacts, let visitors personalize and customize their virtual objects, environments or interfaces or offer interactions like sharing with others that are not afforded by the physical object.

Nevertheless, all interactions between agents, objects and spaces in virtual behaviour settings *can be described* as a set of rules with consequences [6], rendering them commensurate with behaviour setting theory. Neither Newtonian physics nor ‘meatspace’ social interaction rules are ‘baked’ into the behaviour setting concept: all it assumes is the synomorphic operation of environmental, social, psychological and temporal orders. To be clear, we do not imply here that *physical* objects afford and constrain behaviour by executing a rule. They delimit behaviour and setting qua their physical affordances. But *conceptually*, this affordance relation can be described/represented as a rule. The benefit of this kind of analytic redescription is that in virtual behaviour settings, these rules are subject to a mangle or assemblage that involves *code*—that is, ‘physical’ and other orders are constituted and afforded and constrained by software whose control is often out of reach of the immediate interactants in a setting, and software that does indeed constitute rules ontologically as executable algorithms: in virtual 3D spaces, we can point to literal, written rules (algorithms) in the source code that pre-scribe the physical affordances of the space [18,19].

### (b) Embodying an avatar

As an avatar in a virtual environment [20], one’s embodiment is mediated through technology. Forms of embodiment and agency may differ depending on the kinds of sensory input and actuation output provided by the technology, but also on the particular control schemes and realization of one’s presence (e.g. as ‘first person’ or ‘third person’ view). Avatars provide individuals with the opportunity to create and present alternative identities or versions of themselves [21]. This allows for experimentation with different aspects of identity, self-expression and representation, potentially leading to a broadening or modification of one’s self-concept. Interacting with others through avatars can shape how individuals perceive themselves [22,23]. As individuals control and inhabit an avatar, they may develop a sense of ownership and identification with it [24].

### (c) Virtual behaviours

From a behaviour setting viewpoint, the outcome of any setting is the routines that interactants enact—that is, the sequence of behaviours they perform.^4^ A fundamental characteristic of social interaction is the exchange of information, goods or services. Not only do virtual environments reimplement, emulate or simulate real-world economic exchange processes (from Facebook Marketplace and eBay to auction houses in online role-playing games), but they also include underpinning political-economic systems such as schemes for creating and enforcing virtual contracts and agreements between users. They are also being actively explored as ‘virtual petri dishes’ [25–27], that is, for exploration and experimentation. Here, behaviour setting theory offers an interesting entry point into the conditions under which virtual settings offer sufficient ecological validity or mapping to real-world counterparts of scientific interest or how virtual settings produce demand effects [28]. As with virtual spaces and objects, the possibility space and rules for virtual behaviour and its governance are in large parts offloaded into and prescribed by code [19,29], while many of the informal, implicit, moment-to-moment regulatory mechanisms in bodily face-to-face interactions are remediated by explicit systems such as reputation and rating systems [30].

For the regulation and stabilization of routines, behaviour setting theory puts particular emphasis on setting-inherent feedback loops that support reinforced learning: participants learn through observation, instruction, correction and sanction from others about appropriate routines [2]. Again, virtual settings afford and constrain person-to-person feedback via their interfaces and underlying code, including consequences of action, which may be limited to and by the virtual context. They also selectively ‘filter in and out’ or emulate the complex nonverbal cues, physical proximity and direct face-to-face communication of real-world interaction [13]. The (perceived) stakes and real-world implications can differ. This makes virtual behaviour settings an opportune context for playful activity (i.e. skill-learning in a context of reduced threat or mitigated consequences [31]), but also toxic ‘trolling’ [14]. Furthermore, virtual settings can insert many additional automated feedback mechanisms into the environment. These mechanisms can be hidden or opaque to users (who may not perceive that they access different information and action options in their environment from other interactants). Even more complex is the fact that with machine learning, automated feedback systems themselves are subject to (different kinds of) reinforcement learning as they adapt to observed patterns of user behaviour. Again, this ability to adaptively modify the ‘rules of the game’ does not invalidate the utility of behaviour settings theory, but adds an interesting complexity to analyzing virtual settings.

### (d) Accommodating presence and immersion

The concepts of presence and immersion focus on the subjective experiential involvement in a virtual environment [32,33]. Presence refers to the subjective feeling of being ‘present’ in a virtual environment, while immersion refers to the extent to which individuals feel engaged and absorbed in their virtual experience [34,35]. Various aspects such as realistic real-time graphics, spatial audio, responsiveness and intuitive and ‘naturalistic’ user interfaces impact users’ sense of presence and immersion [36], as do forms of avatar embodiment [37].

Presence and immersion highlight psychological and perceptual aspects of virtual environments that can impact behaviour. How can setting theory accommodate such notions of presence or immersion as experiential phenomena, given its (in)famous demotion of psychological factors?

As a starting point, we propose to conceptualize these as types of configurations between a person and their virtual avatar and its context. These new configurations can include

— ‘extended use’ (for relations with a 2D avatar/object/environment)
— ‘manipulation’ (for relations with an MR/VR object/infrastructure)
— ‘embodiment’ (for relations with an avatar in a VR experience).^5^

The phenomenological particularities of individual experience have not been a central concern of behaviour setting studies but to the extent that these new kinds of configurations have behavioural consequences, then they will require fleshing out in future studies of virtual behaviour settings.

## 5. Tools for thinking about virtual behaviour settings

In MR, virtual information is superimposed onto the physical environment or vice versa, never fully severing the individual actor’s embodied presence in a physical setting. Such augmented reality or virtuality can, therefore, be easily handled by behaviour setting theory within its existing conceptual framework, with an extension allowing for *some* agents and objects to be simulated.

However, 2D or VR environments require a different kind of extension: two settings -- operating in parallel in the real world and VR world -- occurring simultaneously, with information and behavioural links between them, facilitated by a device.

We will discuss two tools for representing behaviour setting findings in the form of diagrams. The first is meant to provide an accessible format to capture relevant information about behaviour settings as a summary for primary research and subsequent intervention. The second is meant to offer deeper insight into the relationships between setting elements.

### (a) The behaviour setting canvas

In all cases, it is possible to represent the virtual world easily as an environment with objects, all of which exist only as simulations, with which the agent interacts through some channel for information transfer (text message, spoken word and finger pinch). The only difference is the ontological status of the agent in that virtual world and the spatial quality of that virtual world (2D or 3D). Such situations can be represented for research or design purposes via a tool, the BSC. The BSC acts as a repository for primary research and a mode of documenting the elements of a behaviour setting in a single place. This form of representation is meant to offer an accessible form of engaging with behaviour settings and also acts as a boundary object to facilitate communication between stakeholders with different backgrounds when discussing behaviour settings. The canvas has been widely used across cultural borders (e.g. in more than seven countries) and on dozens of projects focusing on public health and technology innovation, and in other contexts. Included in these variations have been explorations of partial or wholly virtual contexts, which have provided insight into behaviour setting as a virtual experience. Examples include discussions in chat rooms, a transition to virtual assistants rather than human assistants, virtual gaming and more.

**Figure 1.**
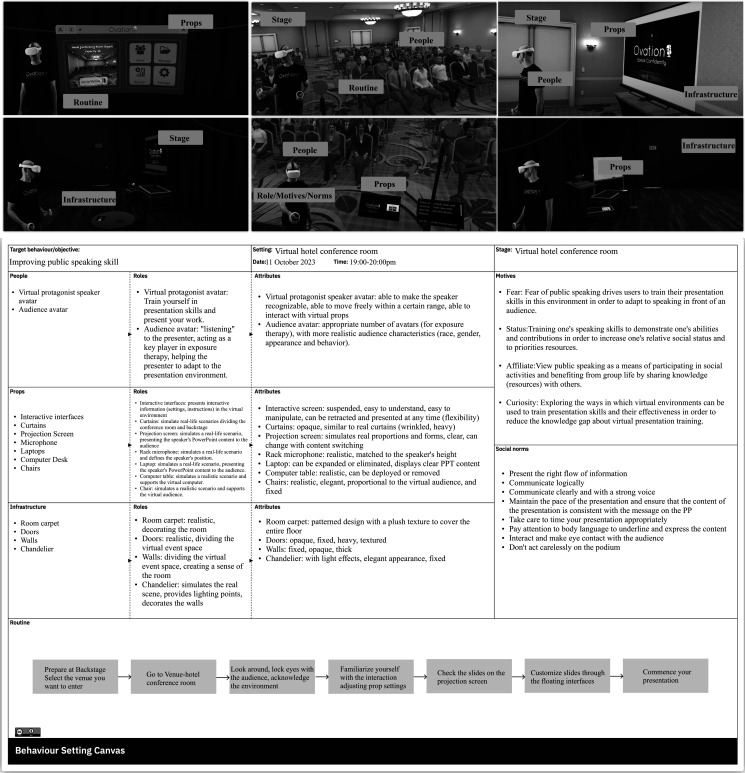
A virtual conference room and (below) associated Behaviour Setting Canvas depicting the setting of public speaking. PPT=Powerpoint.

The canvas enables capturing of the target behaviour, milieu (called ‘stage’) and documentation of research activity (specific setting explored, date and time) across the top of the canvas. The left-hand side of the canvas captures the agents, props and infrastructure along with the associated roles and attributes of these. Importantly, agents, props and infrastructure can be physical or digital elements of the setting in question. The motives and norms found within the setting are noted on the right side of the canvas. Finally, the overall routine (or agent behaviour sequence) is captured across the bottom. The placement of elements on the canvas reflects the practicalities of working and visualizing content and interrelationships rather than any indication of the order in which the content is filled. Indeed, the routine is often the best place to start as it provides an overview. When complete, all sequenced behaviours captured in the routine should also show up in the rest of the canvas in terms of the agent taking action, the props and infrastructure supporting the action and any norms or roles that also influence this. The canvas has been developed as a variation of the behaviour setting checklist found in [5]. Figure 1 shows an example of the BSC applied to a virtual public speaking scenario used for exposure therapy. The images across the top show the virtual conference room setting, and the subsequent canvas below shows the elements as outlined above according to the various elements.

The canvas is useful for both descriptive and prescriptive work. Descriptive work would see it document the existing condition of elements of a behaviour setting and support the researcher in understanding how these elements are interrelated. Prescriptive work explores interventions within the setting by allowing users to focus on a particular element of the canvas and how changes may propagate throughout the setting/canvas. One particularly useful application of the canvas has been in the comparison of existing settings and proposed virtual settings. For instance, if a company plans to use VR for training purposes, it can use the canvas to understand elements in a real setting before translating those into virtual settings. This is one variation of a broader activity afforded by the canvas to understand how to automate or augment technological interventions based on the canvas. As a simple example, consider a person who has the role of introducing individuals at a conference. The attributes (or competencies) needed for this role involve knowing various people and information about them to connect people, and a suggested starting point for a conversation. All of these things can be programmed into technology using a virtual interface for conference participants.

### (b) The behaviour setting model

**Figure 2.**
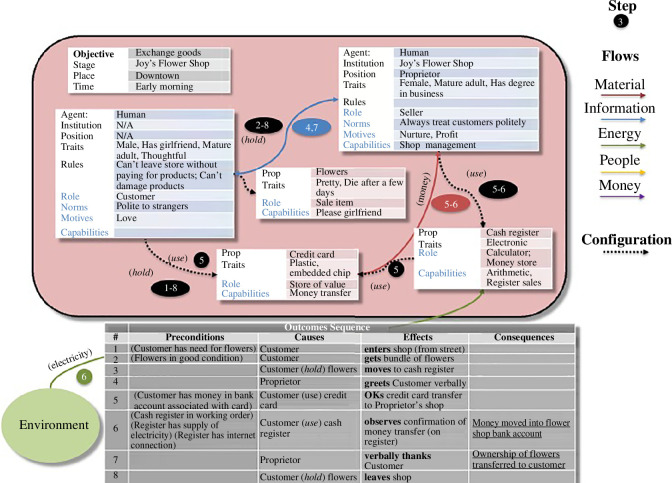
Behaviour setting model of buying flowers.

The behaviour setting model [6], as published (refer figure 2), represents agents, objects (categorized as tools or machines, depending on their complexity and capabilities) and infrastructure as text boxes with sets of characteristics. Primary among these, of course, is the role within the setting, but there are also other (relatively fixed) traits and (more flexible/learned) capabilities. Motives and norms direct role performance, as do the formal constraints (if relevant) of embodying a formal position within some social institution. The behavioural sequence or routine, seen as the outcome of the setting, is represented by a chain of events described in causal terms (in a separate box at the bottom). External forces or phenomena acting on the setting (called ‘context’) have a generic representation as a bubble. Interactions of various kinds (involving ‘flows’ of information exchange, material transfer, etc.) between setting elements are represented by arrows of different colours, which can be restricted to particular events in the outcome sequence (i.e. by event number, represented in a coloured oval). Configurations are (potentially synomorphic) relations between elements of the setting, such as use or make, which help in the performance of actions. Figure 2 represents the commonplace setting of buying flowers in a shop, with no virtual components.

**Figure 3.**
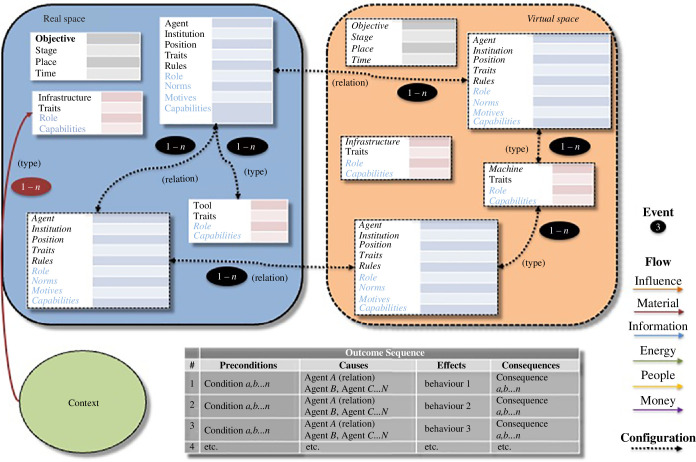
Behaviour setting model (2D and virtual reality).

The model includes facilities for representing virtual elements, however. In a mixed reality scenario, virtual agents and objects can simply be integrated into the model, diagrammatically differentiating them from physical agents and objects with dashed box lines and italicization. Entirely virtual 2D/3D spaces constitute an independent milieu/stage for meaningful activity. Figure 3 shows how the model would represent this via two ‘spaces’, physical and virtual. On the ‘real’ side of the VR setting diagram, you have real-world agents and objects. Typically, at least one of the agents will be interacting with a real-world object that provides access to the virtual world. These activities of the agent can be interactions with virtual objects or agents in the virtual space—a second stage for activities. Indeed, most or all of the important, mission-fulfilling actions may take place in this virtual world in some settings. Physical activities, agents and tools/machines can have virtual counterparts, which are represented via standard (relation) links. Multiple agents can use independent devices from independent spaces to gain access to a common virtual environment, which may multiply the ‘real space’ components of the model.

**Figure 4.**
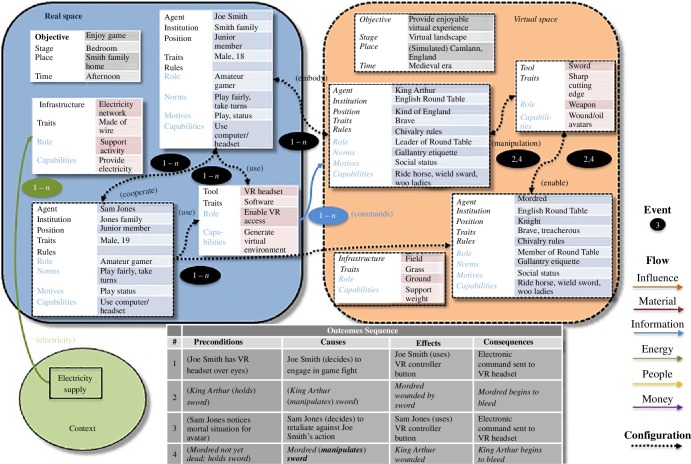
Behaviour setting model example (VR gaming).

An example of VR behaviour is shown (see figure 4) in which two teenagers, using headsets, play a VR game in a shared bedroom, during which they adopt identities (through avatars) as members of the medieval English Round Table (a league of knights defending the island realm). One, however, is a traitor seeking to become king (Mordred), and as a consequence, is attacked by the rightful King Arthur, head of the Round Table, in a field near a town called Camlann, using a sword. The recording of the activity (in the Routine or Outcome Sequence box) switches back and forth between the real-world actions of the teenagers and their virtual counterparts. The activities in the virtual world are a consequence of the type and degree of embodiments and configurations enabled by VR technologies. The example is simplified from an actual implementation, which would be more detailed and include more props and infrastructure, as well as a longer outcome sequence description.

In many games and XR applications, some agents are controlled by the computer, not by human participants. Depending on their concrete implementation and embedding, these may be positioned and diagrammatically modelled as other agents (if they pursue self-directed goals) or machines/tools (if they are inert unless acted on). And just as settings can be structured into several synomorphies, virtual spaces can also be segmented and/or nested within other virtual spaces, e.g. a retail shop within the second-life platform. This can be represented by a virtual space (including agents and infrastructure) literally appearing diagrammatically within or next to another virtual space including agents and other components.

## 6. Discussion

As we hope to have shown, behaviour setting theory does not require much conceptual extension to handle virtual environments, but rather an adaptation of existing conceptual components, notably different implementations of the same components, e.g. extending rule sets to describe the possibilities and consequences of interaction with a type of object as constituted by software, not just physical laws. This could potentially produce differences in kind compared to the physical, mid-20th century settings described by Barker, e.g. new ‘physical’ laws of the virtual environment such as the ability to float in space. Against that stand the facts that 1; behaviour setting theory was originally developed to encompass all the everyday behaviours of whole populations, and so is very general(izable), and 2; people design virtual environments to be ‘natural’, ‘intuitive’ and ‘familiar’, explicitly mirroring and emulating real-world environments. Presence and immersion—the unique aspects of virtual experiences—are not explicitly represented in standard-setting descriptions and thus present a potential genuine addition. But, being subjective, they can often be inferred from the structural contexts within which the agents engage in their role-playing.

While virtual settings present little conceptual challenge to behaviour setting theory, empirically, they do open important questions about whether and how constitutive characteristics of virtual environments systematically moderate the functioning of behaviour settings. Research on whether stratifying characteristics (like gender or body type) evoke the same social responses in virtual avatars as in physical bodies arguably offers a productive template here [24]. Vice versa, work on virtual worlds as digital ‘petri dishes’ for social research [27–29] points to virtual environments as a promising methodological paradigm for behaviour setting research, e.g. replicating and then systematically manipulating or removing individual components of an existing behaviour setting to conduct experimentally the kind of structural and dynamic tests that Barker [1] proposed for identifying behaviour settings and their constituent synomorphies.

On the side of *impact and application*, the behaviour setting concept and related tools (like Model and Canvas) can provide designers with new theory-based aids for the design of virtual environments. Research and practice guidance on how to design virtual environments are arguably fractured into

— *human–computer interaction* and interaction design exploring human factors like cybersickness or the usability of interaction techniques [38];
— *social design* on issues like incentives, reputation systems or community management [39]; and
— ‘industry 4.0’ *VR and digital twinning training and operations* applications concerned with functionally accurate modelling and data flows [40].

Of all these, *game design* (and level design in particular [41]) comes closest to designing for the ways in which virtual environs shape experience. But it is notably concerned with entertainment experiences, not behavioural functions, and focuses on architecture, objects and pre-scripted artificial agent behaviour and does not take social and psychological norm-maintaining mechanisms into account, let alone the functional synomorphy of behaviour-and-milieu components. The primary benefit is that behaviour settings (via Model and Canvas) force designers to consider the full breadth of (physical, psychological, social and temporal) aspects and how they interact with each other to achieve the desired synomorphies that Barker used as a criterion for successful functioning and, by extension, proper design of a setting. This understanding can help shape the layout, interactions and overall design of the virtual environment to optimize user engagement and satisfaction.

## 7. Conclusion

The utility of the behaviour setting concept lies in its unified treatment of heterogeneous elements that interact and are structurally aligned toward some human behaviours and experience, reducing these to a manageable set of elements, whether this experience takes place in a real-world or virtual context. The tools discussed here (setting Canvas and Model) should help developers identify specific correspondences between elements of real and virtual spaces occurring with different levels of immersion. They should also provide a concrete, integrated approach to analyzing the physical, psychological and social aspects of virtual experiences.

Given the likely increase in demand for more immersive virtual experiences (MR and VR) as the technologies for producing such experiences are improved and democratized (in terms of price and ease of use), tools for creating more satisfying immersive experiences should also increase in demand. We argue that the behaviour setting concept, and related models and tools, provide a powerful framework for guiding the development of such experiences through games and other kinds of research and consumer offerings (Zhao [15]). This framework organizes the features of situated experience [6] and hence the dimensions of (designed) experiences, providing a powerful means of building in and checking the necessary features of a rewarding virtual life.

## Data Availability

This article has no additional data.

## Footnotes

^1^
We consider norms and rules not to be rigid, prescriptive algorithmic determinants of behaviour, but rather adaptive/strategic constraints responsive to situational exigencies, designed to achieve the ‘proper’ fulfilment of a role, whatever the circumstances may require.

^2^
This sets the present analysis apart from Blanchard [10], who, to our knowledge, was the first to use the term ‘virtual behaviour setting’ and apply behaviour setting theory to online interactions. Blanchard’s more narrow aim was to conceptualize online communities as virtual behaviour settings constituted by the exchange of communications as the chief virtual object, where ‘place’ is understood to be metaphorical and/or a felt or imagined ‘sense of place’ inhabited by a group of people or their representatives. By contrast, we focus on contemporary multi-user virtual or augmented reality environments as milieus with richly rendered and interactable spaces and objects.

^3^
As noted, experiences only count as taking place in a virtual behaviour setting in our sense if behaviour occurs in a richly realized and experientially circumjacent space with objects. Many computer interfaces present digital ‘spaces’ in a metaphorical sense—interactable surfaces, imagery, text spatially laid out; while these may be synomorphic with a behaviour, they are in the main not experientially circumjacent. We thus analytically exclude any instance where the milieu is experientially not circumjacent or a simple interface metaphor (such as the familiar desktop metaphor) or abstract information space instead of a realized inhabitable space. Certain 2D applications can thus still fit our definition of a virtual behaviour setting *if* they render an actual inhabitable space and interactants can therefore develop a sense of presence and immersion, i.e. that they actually experience themselves to ‘be there’ in a space that surrounds them. *Second Life* accessed through a desktop PC and screen is a virtual behaviour setting in our understanding; typing text in Microsoft Word is not.

^4^
In using the word ‘routine,’ we do not mean to imply that the behavioural sequences resulting from each and every enaction in a setting are without variation. Of course, people respond to unexpected circumstances arising within a setting in strategic ways to ensure their larger goal by the completion of the setting, which may involve a ‘detour’ in terms of actions taken.

^5^
All of these terms have contexts of everyday use that do not refer to virtual experiences according to our definition, for example, in remote surgery, real implements being manipulated can be represented through a 2D analogue on a screen, together with representations of specific real bodies, which one might wish to describe as situations of manipulation and embodiment, but we are advocating a more restricted use of these terms in this context.
